# Complete Genome Sequences of Four Isolates of Vancomycin-Resistant Enterococcus faecium with the *vanA* Gene and Two Daptomycin Resistance Mutations, Obtained from Two Inpatients with Prolonged Bacteremia

**DOI:** 10.1128/MRA.01380-19

**Published:** 2020-02-06

**Authors:** Piroon Jenjaroenpun, Thidathip Wongsurawat, Zulema Udaondo, Courtney Anderson, James Lopez, Meera Mohan, Ruslana Tytarenko, Brian Walker, Intawat Nookaew, David Ussery, Atul Kothari, Se-Ran Jun

**Affiliations:** a Department of Biomedical Informatics, University of Arkansas for Medical Sciences, Little Rock, Arkansas, USA; b Division of Infectious Diseases, University of Arkansas for Medical Sciences, Little Rock, Arkansas, USA; c Myeloma Institute, University of Arkansas for Medical Sciences, Little Rock, Arkansas, USA; University of Rochester School of Medicine and Dentistry

## Abstract

Here, we present complete genome sequences of four Enterococcus faecium isolates, obtained from two patients with apparent vancomycin-resistant Enterococcus faecium bacteremia; these isolates also carried two mutations known to be associated with daptomycin resistance. Sequences were obtained using *de novo* and hybrid assembly of Oxford Nanopore and Illumina sequence data.

## ANNOUNCEMENT

Enterococcus faecium, a Gram-positive opportunistic bacterial pathogen, has become one of the leading causes of nosocomial infections (1). Bacteremia caused by vancomycin-resistant Enterococcus faecium (VREfm) is associated with increased mortality and length of hospital stay.

We previously sequenced 48 VREfm isolates collected from the University of Arkansas for Medical Sciences (UAMS) Hospital using short-read sequencing (2). Here, we report the complete genome sequences of four clinical VREfm isolates. These four VREfm isolates were collected before and after daptomycin treatment from two patients with prolonged bacteremia at the UAMS. Patient 1 was a 63-year-old male with acute myeloid leukemia (AML) who underwent chemotherapy, and patient 2 was a 64-year-old male with myelodysplastic syndrome who underwent a haploidentical stem cell transplant. For whole-genome sequencing using Oxford Nanopore Technologies (ONT) and the Illumina platform, we selected two isolates from each patient, the first and last isolates from an episode of VREfm bacteremia during hospitalization, as follows: two isolates obtained on 28 June 2018 (UAMSEF_01) and 7 July 2018 (UAMSEF_08) from patient 1 and two isolates obtained on 18 September 2018 (UAMSEF_09) and 26 September 2018 (UAMSEF_20) from patient 2.

The VREfm isolates from positive blood cultures were subcultured on blood agar plates. Isolated colonies on the blood agar plates were picked and resuspended into a DNA/RNA Shield collection and lysis tube (Zymo Research, Irvine, CA). Genomic DNA was extracted from each tube using a ZymoBiomics miniprep kit (Zymo Research). Each genomic DNA sample was subdivided into two aliquots; one was subjected to ONT library preparation and the other to Illumina library preparation. The ONT library preparation was performed using a Rapid Barcoding kit (catalog number SQK-RBK004 [ONT]). The barcoded constructed library (all 4 samples) was loaded into an R9.4/FLO-MIN106 flow cell on a MinION device and run for 48 h. ONT raw signals were base called, demultiplexed using Albacore v2.3.4 (ONT), and adapter trimmed with Porechop v0.2.3 (https://github.com/rrwick/Porechop) using default parameters. Following the pipeline used by Jenjaroenpun et al. (3), the reads were filtered by a mean quality score of 9 and a minimum read length of 2,000 bases to retain 0.86, 0.65, 1.33, and 1.11 Gb in total for UAMSEF_01, UAMSEF_08, UAMSEF_09, and UAMSEF_20, respectively. The Illumina library was prepared using a Kapa HyperPlus kit (Roche), and paired-end sequencing was done on the NextSeq 550 platform. We ran fastp v0.19.5 with default parameters (4) to perform quality control, read filtering, and base correction of Illumina reads, retaining 0.77, 0.87, 1.14, and 0.81 Gb in total for UAMSEF_01, UAMSEF_08, UAMSEF_09, and UAMSEF_20, respectively. We performed *de novo* hybrid assembly of Illumina and ONT reads (5) using Unicycler v0.4.4 (6) with default parameters. In the case of a single contiguous circular chromosome not being produced by Unicycler, we first assembled the chromosome using Canu v1.8 (7), then circularized it using Circlator v1.5.5 (8) with modified parameters (–merge_min_id 85 –merge_breaklen 1000 –verbose –assembler canu –split_all_reads –data_type nanopore-raw –bwa_opts “-x ont2d”), and then took two additional steps to improve assembly quality, namely, one round of correction with ONT reads using NanoPolish v0.11.0 (9) and two iterative error corrections with Illumina reads using Pilon v1.22 (10). The quality of genome sequences was checked using QUAST v5.0.2 (11) and annotated by the Prokaryotic Genome Annotation Pipeline (PGAP) (12). The Comprehensive Antibiotic Resistance Database (CARD) (13) was used to complement the detection of antibiotic resistance genes in the assembled genomes.

For patient 1, *de novo* hybrid assembly generated complete circular chromosomes of 2,824,869 bp (G+C content, 38.16%) and 2,827,987 bp (G+C content, 38.16%). Annotated genome assemblies are publicly available in the NCBI database under GenBank accession numbers CP035648 and CP035654, respectively. For patient 2, the assembly of both isolates resulted in complete chromosomes of 2,912,202 bp (G+C content, 38.10%) and 2,912,170 bp (G+C content, 38.10%), available under accession numbers CP035660 and CP035666, respectively. Each isolate contains five plasmids, listed in Table 1. All VREfm isolates carried a transferable plasmid harboring a *vanA* gene. In addition, all VREfm isolates harboring comutations (LiaS^T120A^ and LiaR^W73C^) commonly associated with daptomycin resistance (14) were identified against the CARD database.

**TABLE 1:** Isolate details and associated features

Sample	GenBank accession no.	Type of contig	Total length (bp)	G+C content (%)	No. of predicted ORFs^*a*^	*vanA* present
UAMSEF_01	CP035648	Circular chromosome	2,824,869	38.16	2,800	No
	CP035649	Circular plasmid	257,028	35.71	302	No
	CP035644	Circular plasmid	37,314	35.14	44	Yes
	CP035645	Circular plasmid	6,303	36.09	9	No
	CP035646	Circular plasmid	4,316	36.93	5	No
	CP035647	Circular plasmid	3,030	34.59	4	No
UAMSEF_08	CP035654	Circular chromosome	2,827,987	38.16	2,804	No
	CP035655	Circular plasmid	256,961	35.71	303	No
	CP035650	Circular plasmid	37,293	35.15	44	Yes
	CP035651	Circular plasmid	6,303	36.09	9	No
	CP035652	Circular plasmid	4,316	36.93	5	No
	CP035653	Circular plasmid	3,008	34.64	4	No
UAMSEF_09	CP035660	Circular chromosome	2,912,202	38.10	2,918	No
	CP035661	Circular plasmid	295,052	35.67	351	Yes
	CP035656	Linear plasmid	81,204	33.54	98	No
	CP035657	Circular plasmid	6,303	36.09	9	No
	CP035658	Circular plasmid	4,316	36.93	5	No
	CP035659	Circular plasmid	3,008	34.64	4	No
UAMSEF_20	CP035666	Circular chromosome	2,912,170	38.10	2,918	No
	CP035667	Circular plasmid	294,919	35.66	351	Yes
	CP035662	Linear plasmid	81,272	33.53	98	No
	CP035663	Circular plasmid	6,303	36.09	9	No
	CP035664	Circular plasmid	4,316	36.93	5	No
	CP035665	Circular plasmid	3,008	34.64	4	No

a ORF, open reading frame.

### Data availability.

Accession numbers are listed in Table 1. Raw sequences were deposited into the NCBI SRA database under BioProject number PRJNA518133.
